# Crop cultivation planning with fuzzy estimation using water wave optimization

**DOI:** 10.3389/fpls.2023.1139094

**Published:** 2023-03-06

**Authors:** Li-Chang Liu, Kang-Cong Lv, Yu-Jun Zheng

**Affiliations:** School of Information Science and Technology, Hangzhou Normal University, Hangzhou, Zhejiang, China

**Keywords:** crop cultivation planning, optimization, fuzzy parameters, evolutionary algorithms, water wave optimization (WWO).

## Abstract

In a complex agricultural region, determine the appropriate crop for each plot of land to maximize the expected total profit is the key problem in cultivation management. However, many factors such as cost, yield, and selling price are typically uncertain, which causes an exact programming method impractical. In this paper, we present a problem of crop cultivation planning, where the uncertain factors are estimated as fuzzy parameters. We adapt an efficient evolutionary algorithm, water wave optimization (WWO), to solve this problem, where each solution is evaluated based on three metrics including the expected, optimistic and pessimistic values, the combination of which enables the algorithm to search credible solutions under uncertain conditions. Test results on a set of agricultural regions in East China showed that the solutions of our fuzzy optimization approach obtained significantly higher profits than those of non-fuzzy optimization methods based on only the expected values.

## 1 Introduction

Many agricultural areas have complex and diverse topographic features (Rabia et al., 2022). In an area of one or several square kilometers, soil properties often change greatly, and different soil properties are suitable for different crops (Fu et al., 2023). The planning of crop cultivation in such a complex agricultural area needs to determine the appropriate crop for each plot of land to maximize the expected total profit, which is an important but difficult problem from an agricultural management point of view (Thilakarathne et al., 2023). The problem has to consider many factors including not only the topography and soil properties, but also the investment budget and cost of cultivation, expected yield of each plot, and selling price of each crop. However, the factors such as cost, yield, and selling price are typically uncertain and hard to estimate exactly. How to appropriately characterize these factors becomes a challenging task in the problem formulation.

In this paper, we present a problem of crop cultivation planning that aims to maximize the expected total profit under the constraint of investment budget and potential loss, where the uncertain factors are characterized as fuzzy parameters. Therefore, the problem is formulated as a fuzzy optimization problem, which is much more complex than its crisp counterpart. Classical exact optimization approaches typically use methods such as expected functions and centroids to transform fuzzy values into crisp values, which inevitably lose important information contained in fuzzy parameters (Ekel et al., 1998; Liu, 2002; Zheng and Ling, 2013; Luhandjula, 2015).

To solve the problem more credibly by fully utilizing the information contained in fuzzy parameters, we propose an approach that evaluates the objective function with three metrics based on the expected, optimistic and pessimistic value models developed by Liu (2007). Based on the comprehensive fitness evaluation approach, we adapt an efficient evolutionary algorithm, water wave optimization (WWO) (Zheng, 2015) for the problem, which evolves the solutions to simultaneously improve the fitness in terms of the three related but different metrics. We conduct computational experiments on a variety of test instances constructed on agricultural regions in East China, and the results validate that the solutions obtained by the proposed WWO algorithm with fuzzy optimization obtain significantly higher profits than those of popular non-fuzzy evolutionary algorithms based on only the expected values. The main contributions of this paper can be summarized as follows:

- We present a crop cultivation planning problem that uses fuzzy parameters to characterize uncertain factors.
- We propose an adapted WWO algorithm to efficiently solve the fuzzy optimization problem.
- We validate the proposed method on a variety of test instances.

The remainder of the paper is organized as follows. Section 2 reviews the related work, Section 3 presents the crop cultivation planning problem, Section 4 describes the adapted WWO algorithm for the problem, Section 5 presents the test results, and Section 6 concludes with a discussion

## 2 Related work

Optimization models and algorithms have been widely used in agricultural planning. Zuo et al. (1991) studied a production planning problem for a large seed corn production company in North America in order to minimize the total cost by allocating the production of corn hybrids to different geographical areas; they developed a series of mathematical programming models and proposed a linear programming package and a mixed-integer programming package combined by a designed heuristic program to solve the problem. Sarker et al. (1997) presented a linear programming model for crop planning in Bangladesh that aims to maximize the overall contribution having satisfied the food demand, land availability, and capital constraints. Detlefsen and Jensen (2004) presented a decision support system, which calculates for each variety of winter wheat the expected net revenue as the expected gross revenue minus the expected costs for treatment of diseases and application of additional fertilization; the decision process was represented as a simple stochastic optimization model. Janová (2012) developed specific validation and verification procedures for the crop planning optimization models in agriculture when the randomness of harvests is considered and complex crop rotation restrictions must hold; the procedures were applied to stochastic programming model constructed as a decision support tool for crop plan optimization in South Moravian farm. López-Mata et al. (2016) developed a direct-solution algorithm capable of determining the crop ´ planning (area and volume of water per crop) that maximizes the profitability of an irrigation farm based on the data including the total cultivable area of the farm, the amount of available irrigation water, and the “gross margin vs. irrigation depth” functions of the considered crops. Esteso et al. (2022) presented a centralized multi-objective mathematical programming model to support the sustainable crop planning definition for a region that jointly optimize three objectives including supply chain profits maximization, waste minimization, and unfairness among farmers minimization; the multi-objective model was solved by applying the weighted sum method.

Except the simplest linear programming model, integer, mixed-integer, and multi-objective programming models are all NP-hard, for which exact optimization algorithms are applicable to only small- or medium size problem instances. Many recent efforts have been devoted to evolutionary algorithms for find near optimal or acceptable solutions to complex crop planning problems. Sarker and Ray (2009) formulated a crop-planning problem as a bi-objective optimization model that maximizes the total gross margin while minimizing the total working capital required; they solved two versions of the problem using multi objective evolutionary algorithms. Adeyemo et al. (2010) considered a multi-objective crop planning problem with three objectives including total net benefit maximization, agricultural output maximization, and total irrigation water minimization; they transformed the model into a single-objective one by taking the latter two as constraints, and then solved the single-objective optimization problem using differential evolution (DE) (Storn and Price, 1997). Márquez et al. (2011) modeled a water-saving crop planning problem as a multi-objective optimization problem that not only maximizes the economic benefits but also minimizes the water used; they solved the problem using two multi-objective evolutionary algorithms to search for Pareto-optimal solutions representing a trade-off between the two objectives. The water-saving crop planning problem considered by Wang et al. (2012) used four objective functions including maximum total net output, total grain yield, ecological benefits, and water productivity; they employed a multiple objective chaos particle swarm optimization (PSO) algorithm to solve the problem. Chetty and Adewumi (2014) compared a genetic algorithm (GA) and several swarm intelligence metaheuristics including cuckoo search, firefly algorithm, and glowworm swarm optimization, in solving an NP-hard annual crop planning problem. Zheng et al. (2013) studied a multiobjective oil crop fertilization problem, which takes into consideration not only crop yield and quality but also energy consumption and environmental effects; the authors proposed a hybrid multiobjective fireworks optimization algorithm that evolves a set of solutions to the Pareto optimal front, using the concept of Pareto dominance for individual evaluation and selection. Fereidoon and Koch (2018) used a complex coupled simulation-optimization tool combining constrained PSO and LINGO-sub-optimization to solve crop planning in the Karkheh River Basin, Iran, under the impacts of climate change. Lin et al. (2021) presented a mathematical programming model for annual crop planning that allocates a land area for growing dryland and wetland crops to maximize the total profit and minimize the total irrigation water used for multiple cropping, and they proposed a simplified swarm optimization that improves PSO with four probabilities to determine the operations of updating solutions to effectively solve the problem.

In practice, agricultural systems are related to various uncertainty factors from the environment and market. However, only a few studies formulate these uncertainties into crop planning problems. Niu et al. (2016) developed an interactive two-stage fuzzy stochastic programming method for supporting crop planning and water resource allocation, where uncertainties are expressed as probability distributions and fuzzy-boundary intervals; the method enables decision makers to identify a trade-off between higher objective values and feasibility of constraints, and was applied to a real case of Hetao irrigation district in China. Alemany et al. (2021) developed a set of mathematical programming models to plan the planting and harvest of fresh tomatoes under a sustainable point of view for multi-farmer supply chains under uncertainty in different decision-making scenarios; for each distributed scenario, the individual solution per farmer as regards the planting and harvesting decisions per crop were integrated to obtain the overall supply to satisfy the markets demand. To the best of our knowledge, there are few studies conducted on evolutionary algorithms for solving large-size crop planning problems (typically of tens to hundreds of plots of lands and types of crops) with uncertain factors.

## 3 Problem description

### 3.1 Basic problem formulation

In the considered problem, we have an agricultural region that is divided into a set of *m* plots of lands, denoted by {*P*_1_,*P*_2_,…,*P_m_*}. The area of each plot *P_i_* is *a_i_* hectares; as the topographic conditions and soil properties inside a plot are homogeneous, each plot is allowed to cultivated with only one type of crop in the planning horizon (i.e., a particular season).

There are *n* types of candidate crops, denoted by {*C*_1_,*C*_2_,…,*C_n_*}. If plot *P_i_* is cultivated with crop *C_j_*, the basic investment is *ũ_ij_* (including investment for seeds, pesticides, fertilizers, irrigation, cultivation machines, etc.) per hectare, the expected yield is *g_ij_* kg per hectare, and the cost for harvesting the crop is *_ij_* per kg. The expected selling price of crop *C_j_* (after harvesting) is *p_j_* per kg (1≤*i*≤*m*;1≤*j*≤*n*). The superscript ˜ indicates that due to uncertain conditions, the corresponding variable is difficult to determined exactly, and therefore is estimated as a fuzzy number.

The problem is to determine for each plot *P_i_* the type of crop to be cultivated. Therefore, the decision variables can be expressed by an *m*-dimensional integer vector ***x*** ={*x*_1_,*x*_2_,…,*x_m_*}, where *x_i_* denotes the type of crop in *P_i_*, i.e., *P_i_* is cultivated with crop *C*_*x*_*i*__ (1≤*x_i_* ≤*n*).

Given a solution vector ***x*** ={*x*_1_,*x*_2_,…,*x_m_*}, the expected overall revenue of the cultivation decision can be calculated as:


(1) $$f\left(x\right)=\sum_{i=1}^{m}a_{i}\left({\tilde{g}}_{i,x_{i}}\left({\tilde{p}}_{x_{i}}-{\tilde{v}}_{i,x_{i}}\right)-{\tilde{u}}_{i,x_{i}}\right)$$

The total budget is *B*, and the maximum loss that can be tolerated by the blueinvestor is *L*. Therefore, the budget constraint and loss constraint can be described as follows:


(2) $$\sum_{i=1}^{m}a_{i}\left({\tilde{u}}_{i,x_{i}}+{\tilde{g}}_{i,x_{i}}{\tilde{v}}_{i,x_{i}}\right)\leq B$$

(3) $$\sum_{i=1}^{m}l\left(i,x_{i}\right)\leq L$$

where *l*(*i*,*x_i_*) denotes the loss in plot *P_i_* cultivated with crop *C*_*x*_*i*__ , which is calculated as:


(4) $$l(i,x_{i})=\{\begin{array}{ll} \max(a_{i}{\tilde{u}}_{i,x_{i}}-a_{i}{\tilde{g}}_{i,x_{i}}({\tilde{p}}_{x_{i}}-{\tilde{v}}_{i,x_{i}}),0), & {\tilde{p}}_{x_{i}}\geq {\tilde{v}}_{i,x_{i}}\\ a_{i}{\tilde{u}}_{i,x_{i}}-a_{i}{\tilde{g}}_{i,x_{i}}({\tilde{v}}_{i,x_{i}}-{\tilde{p}}_{x_{i}}) & {\tilde{p}}_{x_{i}}<{\tilde{v}}_{i,x_{i}} \end{array}$$

### 3.2 Evaluation of fuzzy parameters

If all input parameters are crisp values, the above formulation (1)–(4) can be regarded as an exact integer programming model. However, at the beginning of the planning horizon, some important parameters are difficult to estimated accurately. In this work, we express the investment *ũ_ij_*, yield rate *g_ij_*, and harvest cost *_ij_* as interval fuzzy numbers $\left[{\underline{u}}_{ij},{\bar{u}}_{ij}\right]$ , $\left[{\underline{g}}_{ij},{\bar{g}}_{ij}\right]$ , and $\left[linev_{ij},{\bar{v}}_{ij}\right]$ , respectively, where an underline denotes a lower limit and an overline denotes an upper limit (1≤*i*≤*m*, 1≤*j*≤*n*); we express the expected selling price *p_j_* as a Gaussian fuzzy number *N*(*μ_j_*,*σ_j_*), where *μ_j_* is the mean value and *σ_j_* is the deviation (1≤*j*≤*n*). Nevertheless, other types of fuzzy numbers (e.g., triangular and trapezoidal fuzzy numbers) are also allowable in the formulation of our fuzzy optimization problem. The fuzzy values can be estimated from historical data based on regression, fuzzy logic, and other machine learning methods that are capable of modeling uncertainty (Zheng et al., 2017a; Zheng et al., 2017b; Hernández and López, 2020; Gavahi et al., 2021).

As the budget constraint is a hard constraint, we use upper limits of investments and costs to transform the fuzzy constraint (2) as:


(5) $$\sum_{i=1}^{m}a_{i}\left({\bar{u}}_{i,x_{i}}+{\bar{g}}_{i,x_{i}}{\bar{v}}_{i,x_{i}}\right)\leq B$$

For the loss constraint, we evaluate the selling price of crop *C_j_* as *p_j_*–3*σ_j_* (the probability that the selling price is even smaller is less than 0.27% and is therefore negligible); moreover, if this selling price is larger than the harvest cost, we consider the lower limit of yield; otherwise, we consider the upper limit of yield; consequently, equation (4) is transformed as:


(6) $$l'(i,x_{i})=\{\begin{array}{ll} \max(a_{i}{\bar{u}}_{i,x_{i}}-a_{i}{\underline{g}}_{i,x_{i}}(\mu_{x_{i}}-2\sigma_{x_{i}}-{\bar{v}}_{i,x_{i}}),0), & \mu_{x_{i}}-2\sigma_{x_{i}}\geq {\bar{v}}_{i,x_{i}}\\ a_{i}{\bar{u}}_{i,x_{i}}-a_{i}{\bar{g}}_{i,x_{i}}({\bar{v}}_{i,x_{i}}-\mu_{x_{i}}+2\sigma_{x_{i}}), & \mu_{x_{i}}-2\sigma_{x_{i}}<{\bar{v}}_{i,x_{i}} \end{array}$$

Normally, the objective function (1) can be evaluated by using expected values *μ*_*x*_*i*__ , $u_{i,x_{i}}=\left({\underline{u}}_{i,x_{i}}+{\bar{u}}_{i,x_{i}}\right)/2$ , $g_{i,x_{i}}=\left({\underline{g}}_{i,x_{i}}+{\bar{g}}_{i,x_{i}}\right)/2$ , and $v_{i,x_{i}}=\left({\underline{v}}_{i,x_{i}}+{\bar{v}}_{i,x_{i}}\right)/2$ for fuzzy parameters:


(7) $$E\left(x\right)=\sum_{i=1}^{m}a_{i}\left(g_{i,x_{i}}\left(\mu_{x_{i}}-v_{i,x_{i}}\right)-u_{i,x_{i}}\right)$$

However, in practice, the expected value could deviate largely from the actual value. Therefore, we employ a credibility model (Liu, 2007) that calculates a credibility value *C_r_* (*ξ*) for a fuzzy variable *ξ* with membership function *μ* over the base set *B* of real numbers as follows:


(8) $$C_{r}\left\{\xi \in B\right\}=\left(\underset{\xi \in B}{\sup}\mu \left(x\right)+1-\underset{\xi \in B^{\text{C}}}{\sup}\mu \left(x\right)\right)/2$$

Given a confidence *θ*∈(0,1], the *θ*-optimistic value and *θ*-pessimistic value of *ξ* are respectively defined as follows:


(9) $$O\left(\xi ,\theta \right)=\sup\;\{r|C_{r}\left\{\xi \geq r\right\}\geq \theta \}$$

(10) $$P\left(\xi ,\theta \right)=\inf\;\{r|C_{r}\left\{\xi \geq r\right\}\leq \theta \}$$

Based on the model, we also respectively evaluate an optimistic objective value and a pessimistic objective value as follows:


(11) $$O\left(x,\theta \right)=\sum_{i=1}^{m}a_{i}\left(O\left(g_{i,x_{i}},\theta \right)\left(O\left(p_{x_{i}},\theta \right)-O\left(v_{i,x_{i}},\theta \right)\right)-O\left(u_{i,x_{i}},\theta \right)\right)$$

(12) $$P\left(x,\theta \right)=\sum_{i=1}^{m}a_{i}\left(P\left(g_{i,x_{i}},\theta \right)\left(P\left(p_{x_{i}},\theta \right)-P\left(v_{i,x_{i}},\theta \right)\right)-P\left(u_{i,x_{i}},\theta \right)\right)$$

where the parameter *θ* is specified by the decision maker. The three objective values {*E*(***x***), *O*(***x***,*θ*), *P*(***x***,*θ*)} constitute a comprehensive evaluation of fitness of each solution ***x*** to the fuzzy optimization problem.

If a solution violates the constraints, we calculates the violation degree as:


(13) $$v\left(x\right)=\max\;(\sum_{i=1}^{m}a_{i}\left({\bar{u}}_{i,x_{i}}+{\bar{g}}_{i,x_{i}}{\bar{v}}_{i,x_{i}}\right)-B,0)+\max\;(\sum_{i=1}^{m}l'\left(i,x_{i}\right)-L,0)$$

And then the objective function of the solution is added by a penalty of *Mv*(***x***), where *M* is a large positive number.

## 4 Water wave optimization for the problem

### 4.1 Basic water wave optimization

**Figure 1:**
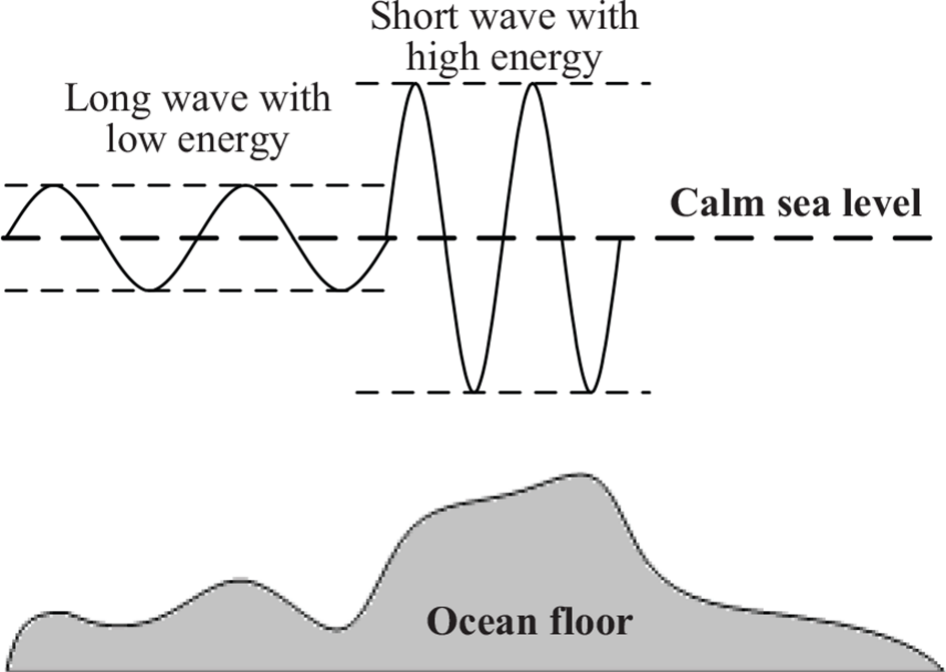
Illustration of wave heights and lengths in shallow water.

WWO is a relatively new evolutionary algorithm inspired by the shallow water wave theory (Zheng, 2015), where the solution space is analogous to a seabed area, each solution ***x*** is analogous to a water wave associated with a wavelength *bda_x_*, and the fitness of a solution is measured inversely according to its seabed depth. According to the shallow water wave theory, the shorter the distance between the seabed and the wave, the higher the wave height is and the smaller the wave length is, as illustrated in **Figure 1**.

At each iteration of the WWO algorithm, each wave ***x*** propagates in a range proportional to its wavelength, such that better solutions exploit smaller areas and worse solutions explore larger areas to balance between the local and global search to generate new solutions. In a high-dimensional continuous solution space, the propagation operation is executed by shifting each dimension *i* of the ***x*** as follows:


(14) $$x_{i}=x_{i}+rand\left(-1,1\right)\cdot \lambda_{x}L_{i}$$

where *rand* is a function that generates a uniformly distributed random number within the specified range, and *L_i_* is the length of the *i*th dimension of the solution space.

All wavelengths are initialized to 0.5 and then updated based on solution fitness at each iteration as follows:


(15) $$\lambda_{x}=\lambda_{x}\alpha^{-(f(x)-f_{\min}+\epsilon )/(f_{\max}-f_{\min}+\epsilon )}$$

where *f*_max_ and *f*_min_ are the maximum and minimum fitness values among the population, respectively, *ϵ* is a very small value to avoid the zero-division error, and *α* is a parameter for wavelength reduction.

In addition to propagation, the basic WWO have two other operators: refraction and breaking. The refraction operator performs on any wave ***x*** that has not been improved after a certain number of generations by learning from the current best solution x^*^ at each dimension *i* as follows:


(16) $${x^{\prime }}_{i}=N\left(\frac{x_{i}^{*}+x_{i}}{2},\frac{\left|x_{i}^{*}-x_{i}\right|}{2}\right)$$

where *N*(*μ*,*σ*) generates a Gaussian random number with mean *μ* and standard deviation *σ*. After refraction, its wavelength is updated according to the ratio between the new and original fitness values as:


(17) $$\lambda_{x^{\prime }}=\lambda \frac{f\left(x\right)}{f\left(x^{\prime }\right)}$$

The breaking operator is used to split a newly found best wave ***x*** ^*^ into a series of solitary waves, each of which is obtained by randomly selecting *k* dimensions (where *k* is a random number between 1 and a predefined upper limit *k*_max_) and at each dimension *i* updating the component as:


(18) $${x^{\prime }}_{i}=x_{i}^{*}+rand\left(0,1\right)\cdot \beta L_{i}$$

where *β* is a parameter of breaking range. If the fittest one among the solitary waves is better than x^*^, it will replace x^*^ in the population.

In brief, propagation is the basic search mechanism for balancing global exploration and local exploitation, refraction helps stagnant waves to escape from local optima and improves the diversity of the population, while breaking further enhances the local search ability. The combination of these three operators makes WWO efficient in search in a high-dimensional solution space. Algorithm 18 presents the basic WWO algorithm framework.

The basic WWO algorithm is proposed for continuous optimization problems. Zheng et al. (2019) presented a systematic approach for adapting WWO to various combinatorial optimization problems. The key idea is to define a neighborhood search operation based on a neighborhood structure of the problem, and conduct the propagation on an solutions as a series of steps of neighborhood search, while the number of steps depends on the fitness or wavelength of the solution.

Since its proposal, WWO has attracted considerable attention in both academic and industrial communities. There have been a lot of work on modified WWO algorithms (Zheng and Zhang, 2015; Wu et al., 2017; Zhang et al., 2018; Zhang et al., 2019) and their applications to a variety of engineering optimization problems (Zheng et al., 2017c; Fard and Hajaghaei-Keshteli, 2018; Shao et al., 2018; Shao et al., 2019; Zhao et al., 2019; Zhou et al., 2019; Yan et al., 2021; Su et al., 2022; Zhang et al., 2022).

### 4.2 Adapted water wave optimization for fuzzy optimization

The basic WWO algorithm is for crisp optimization problems. To handle the presented fuzzy crop planning problem, we adapt the WWO in the following aspects.

- Instead of the single current best x^*^ in WWO, the adapted WWO keeps three current bests $x_{E}^{*}$ , $x_{O}^{*}$ , and $x_{P}^{*}$ that have the best expected, optimistic, and pessimistic objective function values found so far, respectively.

Algorithm 1 Basic WWO algorithm. 
1 Randomly initialize a population of NP solutions (waves);
2 Calculate the fitness of each solution, and let x* be the fittest one in the population;
3 **while** *the termination condition is not satisfied* **do**
4       **foreach** *wave x in the population do*
5              **foreach** dimension i **do**
6                      Update xi according to Eq. (14);
7              Let x' be the propagated wave;
8              **if** f(x') > f(x) **then**
9                      Replace x with x';
10                    **if** f(x) > f(x*) **then**
11                            Set x* to x;
12                            **for** *k* = 1 to *rand*(1, *k_max_
*) **do**
13                                                   Select a random dimension i and create a solitary wave x' according to Eq. (18);
14                                  *if* f(x') > f(x*) **then**
15                                          Set x* to x';
16              **else**
17                      **if** x *has not been improved for h^max^ iterations* **then**
18                                       Refract x to a new x' according to Eq. (16);
19                           Update λ_x'_according to Eq. (17);
20       Update the wavelengths of the solutions according to Eq. (15);
21 **return** the best wave found so far.

- At each iteration, let *E*_max_, *O*_max_, and *P*_max_ be the maximum expected optimistic, and pessimistic objective function values in the population, respectively, and *E*_min_, *O*_min_, and *P*_min_ be the corresponding minimum objective function values; for each solution ***x*** in the population, we select the maximum value among (*E*(***x***)–*E*_min_+*ϵ*)/(*E*_max_–*E*_min_+*ϵ*), (*O*(***x***,*θ*)–*O*_min_+*ϵ*)/(*O*_max_–*O*_min_+*ϵ*), and (*P*(***x***,*θ*)–*P*_min_+*ϵ*)/(*P*_max_–*P*_min_+*ϵ*) as the exponent *r*, and update its wavelength as *λ_x_* =*λ_x_α* ^–^ *^r^*.
- When performing a propagation operation on a solution ***x***, each component *x_i_* has a probability of *λ_x_* of being changed to a new value *x_i_*, which is determined by randomly selecting two other solutions, and then set to the corresponding component of the better one.
- A propagated solution ***x’*** will replace its original solution ***x*** if any of the following conditions is satisfied:

1. ***x’*** is a feasible solution, while ***x*** is an infeasible solution;
2. Both ***x’*** and ***x*** are feasible; ***x’*** is better than ***x*** in one objective functions and is not worse than ***x*** in either of the other two objective functions;
3. Both ***x’*** and ***x*** are feasible; ***x’*** is better than ***x*** in two or three objective functions.

- When performing a breaking operation on a solution ***x***, each solitary wave is obtained by selecting a random dimension *i* and setting *x_i_* to a value, which, among all values in [1,*n*], leads to the best improvement (in any of the three objective functions).
- The refraction operator is removed, and the removal of stagnant solutions is done by iteratively reducing the population size from an upper limit $N_{P}^{\max}$ to a lower limit $N_{P}^{\min}$ , as suggested by Zheng and Zhang (2015).

In this way, the population evolves the solutions to improve the fitness in terms of the expected, optimistic, and pessimistic objective function values simultaneously. Finally, the three best solutions $x_{E}^{*}$ , $x_{O}^{*}$ , and $x_{P}^{*}$ are returned to the decision maker for selection.

Algorithm 2 WWO algorithm adapted for the fuzzy crop planning problem. 
1 Randomly initialize a population of NP solutions (waves);
2 Calculate the fitness of each solution, and let 
${\text{x}}_{E}^{*}$
, 
${\text{x}}_{O}^{*}$
 and 
${\text{x}}_{P}^{*}$
 be the solutions with the best expected,optimistic, and pessimistic objective function values, respectively;
3 **while** *the termination condition is not satisfied* **do**
4       **foreach** *wave x in the population* do
5            **foreach** *dimension i* **do**
6                  Update x*
_i_
* according to Eq. (14);
7            Let x' be the propagated wave;
8            **if** x' *is better than x in terms of the comprehensive comparison of the three objective functions* **then**
9                  Replace x with x';
10                      **if** E(x) > E(x^*^) or O(x, θ) > O(x^*^, θ) or P(x, θ) > P(x^*^, θ) **then**
11                         Update the corresponding best solution;
12                         **for** *k* = 1 to *rand*(1, *k_max_
*) **do**
13                                       Select a random dimension i and create a solitary wave x' according to Eq. (18);
14                               **if** x' *leads to a new best solution* **then**
15                                         Update the corresponding best solution;
16         **else**
17                      **if** x *has not been improved in any objective function for h^max^ iterations* **then**
18                                        Refract x to a new x' according to Eq. (16);
                                 Update λx' according to Eq. (17);
20       Update the wavelengths of the solutions;
21 **return** the best wave found so far.

Compared to the basic WWO, the fuzzy WWO algorithm increases the time complexity in two aspects: (1) each solution is evaluated based on the three (related) objective functions; (2) the comparison of each pair of solutions is based on the three objective functions, at least once and at most three times. Consequently, the time complexity of the fuzzy WWO algorithm is at most triple that of the basic WWO.

## 5 Results

We applied the proposed algorithm to six selected agricultural regions in Zhejiang Province, East China. These regions were with different numbers of plots, crops, budgets, and allowable losses, as summarized in **Table 1**. The planning horizon was three months. The investment and cost are measured in RMB yuan.

**Table 1:** Basic information of the six agricultural regions for the applications of the proposed algorithm.

#Region	Area (hectares)	*m* (plots)	*n* (crops)	*B* (RMB Yuan)	*L* (RMB Yuan)
1	471	27	36	550,000	100,000
2	665	39	33	720,000	180,000
3	729	49	39	800,000	160,000
4	1202	76	36	1,200,000	300,000
5	1530	93	41	1,800,000	450,000
6	2808	121	39	2,400,000	500,000

After solving each problem instance, we presented the results to the decision-maker for selection, and obtained the actual total profit of the crop cultivation after the planning horizon. For comparison, we also implemented three evolutionary algorithms, including differential evolution (DE) (Omran and Engelbrecht, 2007), biogeography-based optimization (BBO) (Simon, 2008; Wang and Wu, 2014), and the basic WWO, to solve the crop planning problem by only maximizing the expected objective function (7). We executed each algorithm for 20 runs, and take the best solution among the 20 runs. The profit of each solution is evaluated based on the expected yields of the solution and actual costs and prices at the end of the planning horizon.

**Figure 2:**
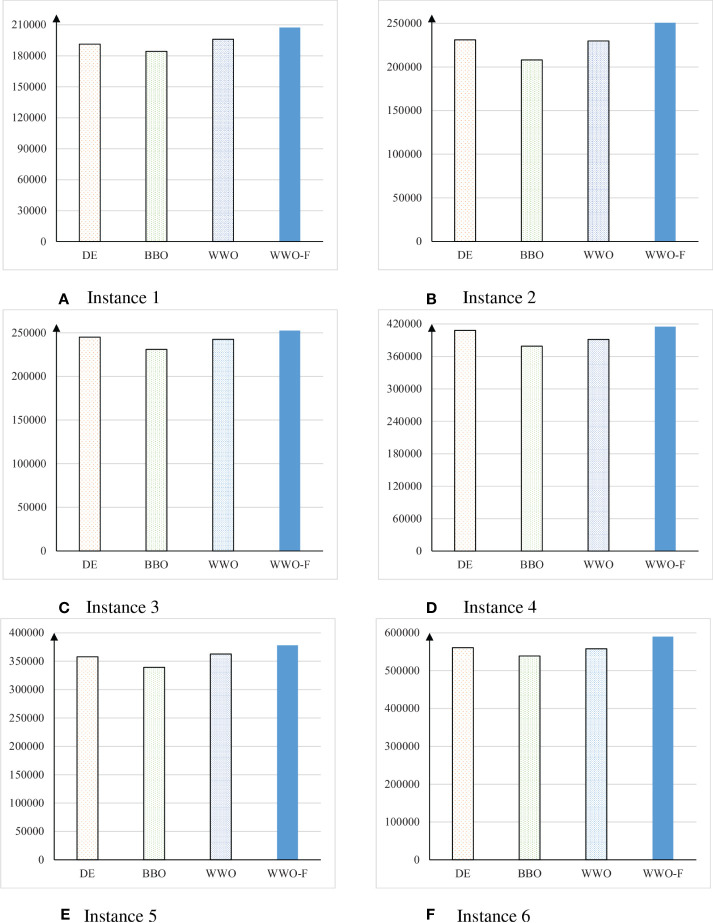
Comparison of the profits of the solutions obtained by the three non-fuzzy evolutionary algorithms and the proposed WWO with fuzzy optimization (WWO-F) on the six test instances **(A–F)**.

**Table 2** presents the profits of the solutions obtained by the different algorithms on the instances, which are also compared in **Figure 2**. On instance 1, WWO solution obtained the maximum profit of 196,009 among the three non-fuzzy evolutionary algorithms, while WWO-F solution obtained a profit of 207,355, which was 11,346 more than maximum profit of the non-fuzzy algorithms. On instance 2, DE solution obtained the maximum profit of 231,060 among the three non-fuzzy evolutionary algorithms, while WWO-F solution obtained a profit of 250,700, which was 19,640 more than that of DE solution. On instance 3, DE solution obtained the maximum profit of 244,960 among the three non-fuzzy evolutionary algorithms, which was 7,640 less than the profit of 252,600 obtained by the WWO-F solution. On instance 4, DE solution obtained the maximum profit of 408,100 among the three non-fuzzy evolutionary algorithms, which was 7,100 less than the profit of 415,200 obtained by the WWO-F solution. On instance5, WWO solution obtained the maximum profit of 362,590 among the three non-fuzzy evolutionary algorithms, which was 15,510 less than the profit of 378,100 obtained by the WWO-F solution. On instance 6, DE solution obtained the maximum profit of 560,800 among the three non-fuzzy evolutionary algorithms, which was 29,400 less than the profit of 590,200 obtained by the WWO-F solution. The results show that, on all six instances, WWO-F always obtained a better profit than the non-fuzzy evolutionary algorithms. This is because the non-fuzzy evolutionary algorithms use only the expected objective function to evaluate the solution fitness; however, the estimated cost and selling price could deviate from the actual values, and hence a solution for maximizing the expected objective function often failed to fully utilize the budget to pursue the maximum profit. By simultaneously using the three criteria including expected, optimistic, and pessimistic values, WWO-F utilized the information contained in the fuzzy parameters much better than the non-fuzzy algorithms, evolved the solutions to keep a good trade-off between the overestimation of the profit and underestimation of the costs, and hence obtained solutions that are more robust and credible.

**Table 2:** Profits of the solutions obtained by the three non-fuzzy evolutionary algorithms and the proposed WWO with fuzzy optimization (WWO-F).

#Region	DE	BBO	WWO	WWO-F
1	191275	184320	196009	207355
2	231060	208040	229750	250700
3	244960	231000	242580	252600
4	v408100	379100	391600	415200
5	357750	339120	362590	378100
6	560800	538800	557900	590200
Total	1993945	1880380	1980429	2094155

The last row of **Table 2** summarizes the total profits of the algorithms on the six instances. DE obtained the maximum total profit of 1,993,945 among the three non-fuzzy evolutionary algorithms, while WWO-F obtained a total profit of 2,094,155, which was 102,210 more than the DE solution. In summary, the proposed fuzzy optimization approach obtained an over five percent increase over the best non-fuzzy algorithm. This result demonstrated the significant economic benefits brought by the application of our fuzzy optimization approach for crop planning.

## 6 Conclusion

This paper presents a crop cultivation planning problem with fuzzy parameters (including cost, yield, and selling price) for maximizing the expected total profit under the constraint of investment budget and potential loss. To fully utilize the information contained in fuzzy parameters, we evaluate the objective function with three metrics based on the expected, optimistic and pessimistic value models, and propose an adapted WWO algorithm that evolves the solutions to simultaneously improve the fitness in terms of the three related but different metrics. Results on a variety of test instances constructed on agricultural regions in East China validated that the solution of the proposed WWO algorithm with fuzzy optimization obtained an over five percent increase on the total profit over the best non-fuzzy algorithm.

The current work studies crop planning in a particular season. Currently, we are extending the fuzzy optimization problem and algorithm for annual crop planning, which involves cultivating and harvesting multiple crops with different seasonal lengths in a plot. Moreover, in the current study, the fuzzy parameters are mainly estimated based on experience or simple regression on historical data; in future work, we will estimate the parameters from big data, using fuzzy deep learning to discover highly nonlinear relationship with complex factors (Song et al., 2019; Elavarasan and Durai Raj Vincent, 2021) and employing transfer learning to utilize knowledge in similar domains to cope with the insufficiency of labeled data (Song et al., 2021; Song et al., 2022; Zheng et al., 2022) in a more comprehensive manner.

## Data availability statement

Publicly available datasets were analyzed in this study. This data can be found here: http://compintell.cn/en/dataAndCode.html.

## Author contributions

L-CL: Investigation, Software, Writing – Original draft preparation. K-CL: Data Curation, Validation. Y-JZ: Conceptualization, Methodology, Funding acquisition. All authors contributed to the article and approved the submitted version.
